# Early Positive Fluid Balance Associates with Increased Mortality in Neurological Critically Ill Patients: A 10-Year Cohort Study

**DOI:** 10.3390/jcm14155518

**Published:** 2025-08-05

**Authors:** Dae Yeon Kim, Sung-Jin Lee, Sook-Young Woo, Jeong-Am Ryu

**Affiliations:** 1 Department of Integrated Internal Medicine Myongji Hospital, Goyang 10475, Republic of Korea; 127avenue@gmail.com; 2 Division of Pulmonary and Critical Care Medicine, Department of Internal Medicine, Ewha Womans University Seoul Hospital, Ewha Womans University College of Medicine, Seoul 07804, Republic of Korea; sungjin.lee@ewha.ac.kr; 3 Biomedical Statistics Center, Research Institute for Future Medicine, Samsung Medical Center, Seoul 06351, Republic of Korea; wsy.woo@samsung.com; 4 Department of Critical Care Medicine, Samsung Medical Center, Sungkyunkwan University School of Medicine, Seoul 06351, Republic of Korea; 5 Department of Neurosurgery, Samsung Medical Center, Sungkyunkwan University School of Medicine, Seoul 06351, Republic of Korea

**Keywords:** critical care, fluid therapy, neurosurgical procedures, hospital mortality, intensive care units

## Abstract

**Background**: Fluid management is a critical aspect of care for neurocritically ill patients, yet the optimal approach remains unclear. The relationship between fluid balance and clinical outcomes in these patients requires further investigation, particularly regarding the timing and volume of fluid administration. **Methods**: This retrospective observational study analyzed 2186 adult patients admitted to the neurosurgical intensive care unit (ICU) from January 2013 to December 2022. We employed a generalized additive model (GAM) with cubic spline smoothing to examine non-linear relationships between fluid balance and mortality. The maximally selected rank statistics method was used to determine the optimal cutoff value for fluid balance. Associations between fluid balance patterns and 28-day mortality were analyzed using a multivariable logistic regression model. **Results**: Initial analysis identified fluid balance on day 1 as the most significant predictor of mortality; patients with positive fluid balance showed a higher 28-day mortality. Non-survivors showed significantly higher fluid input throughout the 7-day observation period, particularly during the first 24 h (4444 mL vs. 3978 mL, *p* = 0.007). Multivariable analysis confirmed that fluid balance on day 1 remained independently associated with 28-day mortality after adjusting for confounders (adjusted odd ratio 1.705, 95% confidence interval: 1.001–2.905, *p* = 0.049). Additionally, the relationship between fluid input day 1 and mortality demonstrated a progressively increasing probability of 28-day mortality with higher fluid volumes. Early fluid balance, particularly during the first 24 h of ICU admission, shows a significant association with mortality in neurocritically ill patients. **Conclusions**: These findings emphasize the crucial importance of careful fluid management in the early phase of neurocritical care and suggest that implementation of strict fluid monitoring protocols, especially during the initial period of care, may improve patient outcomes.

## 1. Introduction

Fluid management is a critical aspect of care for neurocritically ill patients, presenting a complex challenge for clinicians in neurocritical care units. The delicate balance of fluid administration in these patients can significantly impact clinical outcomes, making it a subject of increasing interest and research in the field of neurocritical care [1]. The management of fluid balance in patients with acute brain injury is particularly crucial due to the unique physiological challenges presented by neurological conditions. Excessive fluid intake can lead to serious complications, including pulmonary edema, which can compromise respiratory function and oxygenation [2]. Moreover, in the context of impaired cerebral autoregulation often seen in acute brain injuries, fluid overload may exacerbate brain edema, potentially leading to increased intracranial pressure (ICP) and further neurological deterioration [3]. Conversely, inadequate fluid volume can be equally detrimental. Hypovolemia in neurocritically ill patients may result in compromised cerebral perfusion, potentially leading to brain ischemia and exacerbating secondary brain injury [4]. This highlights the importance of maintaining an optimal fluid balance to ensure adequate organ tissue perfusion and oxygenation, particularly in the vulnerable brain tissue of these patients [5].

Despite the recognized importance of fluid management in neurocritical care, there remains a lack of consensus on the optimal approach to fluid balance in neurological critically ill patients. The specific strategies for maintaining appropriate organ tissue perfusion and oxygenation through fluid management in this patient population are not well established [6]. This gap in knowledge presents a significant challenge for clinicians and underscores the need for further research in this area. Therefore, the objective of this study is to investigate whether early fluid balance patterns and management strategies are associated with clinical outcomes in neurological critically ill patients.

## 2. Materials and Methods

### 2.1. Study Population

**Figure 1:**
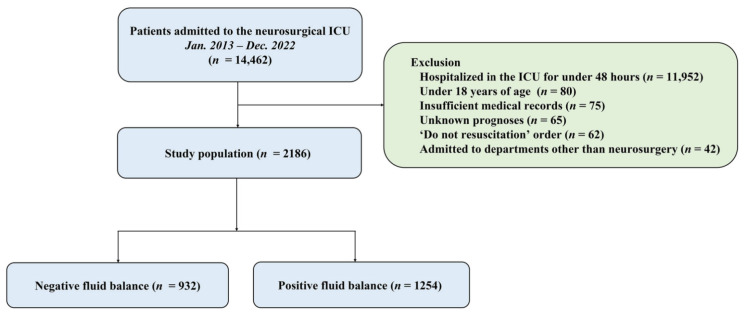
Study flow chart. ICU, intensive care unit.

This retrospective observational study was carried out at a single center, the Samsung Medical Center, a tertiary, referral hospital in Seoul, Republic of Korea. The study included adult patients admitted to the neurosurgical intensive care unit (ICU) from January 2013 to December 2022. The Institutional Review Board (IRB) of Samsung Medical Center granted approval for this study (approval number SMC 2024-02-044). Given the retrospective nature of the research, the IRB waived the requirement for informed consent. The study included neurosurgical patients who were hospitalized in the ICU for more than 48 h. We excluded patients under 18 years of age, those with incomplete medical records, a “do not resuscitate” order, admissions to departments other than neurosurgery, transfers to other hospitals, or those with an uncertain prognosis (Figure 1).

### 2.2. Definitions and Outcomes

In this study, we retrospectively collected baseline characteristics such as comorbidities, behavioral risk factors, ICU management, and laboratory data, utilizing our center’s “Clinical Data Warehouse Darwin-C”. This data warehouse is specifically designed to enable investigators to search and retrieve de-identified medical records from electronic archives. Fluid balance was calculated as the difference between fluid input (all intravenous fluids including any crystalloid, hyperosmotic, or colloid fluids, blood products, enteral fluids, and renal replacement therapy fluids) and fluid loss (urine output, enteral losses, drain losses, and dialysis effluent–dialysate from continuous renal replacement therapy) per day in the ICU. However, insensible fluid losses were not considered [7]. We calculated the mean daily fluid balance and mean daily fluid input. Cumulative fluid input was requested, including fluids that were given in the operating room on days 1–7. The Acute Physiology and Chronic Health Evaluation (APACHE) II score was determined using the worst values recorded in the initial 24 h of ICU admission [8,9]. For intubated patients, the verbal component of the Glasgow Coma Scale (GCS) was estimated based on the eye and motor scores, as previously described [10]. Ventilator-free days were calculated as a composite outcome measure representing the number of days a patient was alive and free from mechanical ventilation within 28 days of ICU admission [11]. For patients who survived and were successfully weaned from mechanical ventilation within 28 days, ventilator-free days were calculated as 28 minus the number of days on mechanical ventilation. Patients who died within 28 days or remained on mechanical ventilation at day 28 were assigned a ventilator-free days score of 0 [11]. The primary endpoint of this study was the 28-day mortality rate.

### 2.3. Statistical Analyses

In this study, continuous variables were reported as medians with interquartile ranges (IQRs). Categorical variables were presented as counts and percentages. For data comparison, we employed the Mann–Whitney U test for continuous variables and the Chi-square test or Fisher’s exact test for categorical variables, as appropriate. The non-linear association between fluid balance and 28-day mortality was examined using a generalized additive model (GAM) with cubic spline smoothing. Additionally, we plotted the association between fluid input and the predicted probability of 28-day mortality, as well as the association between daily fluid balance and the predicted probability of 28-day mortality, using cubic splines in GAM. To determine the optimal cutoff value for fluid balance that could be associated with 28-day mortality, we utilized the maximally selected rank statistics method [12]. To assess the independent association between fluid balance and 28-day mortality, we performed multivariable logistic regression analyses. The relationship between daily fluid balance and 28-day mortality were presented as odds ratios (OR) with 95% confidence intervals (CIs) in a forest plot. *p* values were corrected using Bonferroni’s method to adjust inflated type I error due to multiple testing. All statistical tests were two-sided, with a significance threshold set at a *p* value of less than 0.05. All statistical analyses were conducted using R Statistical Software (version 4.2.0, R Foundation for Statistical Computing, Vienna, Austria).

## 3. Results

### 3.1. Baseline Characteristics of the Study Population

In this study, we analyzed a cohort of 2186 patients (Figure 1). The median age was 57.0 years (IQR: 45.0–68.0) and males constituted 49.0% of the population. The approach using maximally selected rank statistics identified fluid balance on day 1 of 0 mL as the optimal cutoff point, which was subsequently used to categorize patients into negative and positive fluid balance groups. Thus, patients were categorized into two groups based on their fluid balance on day 1—negative fluid balance (*n* = 932) and positive fluid balance (*n* = 1254). The most prevalent comorbidities were malignancy (60.2%), cerebrovascular disease (43.2%), and hypertension (17.0%). Patients in the positive fluid balance group were slightly older (median 58.0 vs. 56.0 years, *p* = 0.003) and had a higher prevalence of cerebrovascular disease (46.6% vs. 38.6%, *p* < 0.001) compared to the negative fluid balance group. Conversely, the negative fluid balance group had a higher prevalence of malignancy (64.6% vs. 56.9%, *p* < 0.001). The leading cause of ICU admission was brain tumor management (47.2%), followed by intracerebral hemorrhage (13.0%) and subarachnoid hemorrhage (10.7%). The positive fluid balance group showed higher proportions of patients admitted for intracerebral hemorrhage (14.7% vs. 10.8%) and traumatic brain injury (9.6% vs. 7.5%) compared to the negative fluid balance group (*p* = 0.001). Patients in the positive fluid balance group presented with a higher APACHE II score at ICU admission (median 11.0 vs. 10.0, *p* < 0.001). While mechanical ventilation use was similar between groups (49.3% vs. 45.7%, *p* = 0.107), the duration was significantly longer in the positive fluid balance group (median 7.0 vs. 3.0 days, *p* < 0.001). The use of mechanical ventilation was similar between groups (49.3% vs. 45.7%, *p* = 0.107), but the duration of mechanical ventilation was longer in the positive fluid balance group (median 7.0 vs. 3.0 days, *p* < 0.001). Interestingly, while the use of ICP monitoring was similar between groups (32.2% vs. 34.1%, *p* = 0.374), the negative fluid balance group had a slightly higher proportion of patients undergoing this procedure (Table 1).

### 3.2. Association Between Fluid Balance and Clinical Outcomes

Analysis of fluid management patterns revealed significant differences between survivors and non-survivors. Fluid balance on day 1 was significantly higher in non-survivors than in survivors (480.0 mL [−104.0–865.5] vs. 156.0 mL [−432.5–763.5], *p* = 0.042). Daily fluid input was consistently higher in non-survivors throughout the 7-day period, with particularly notable differences in the early period (day 1: 4444.0 mL vs. 3978.0 mL, *p* = 0.007). Mean fluid input up to the first three days (4689.0 mL vs. 3905.3 mL, *p* < 0.001), five days (4644.6 mL vs. 3705.2 mL, *p* < 0.001), and seven days (4348.6 mL vs. 3513.2 mL, *p* < 0.001) remained significantly higher in non-survivors compared to survivors (Table 2).

**Figure 2:**
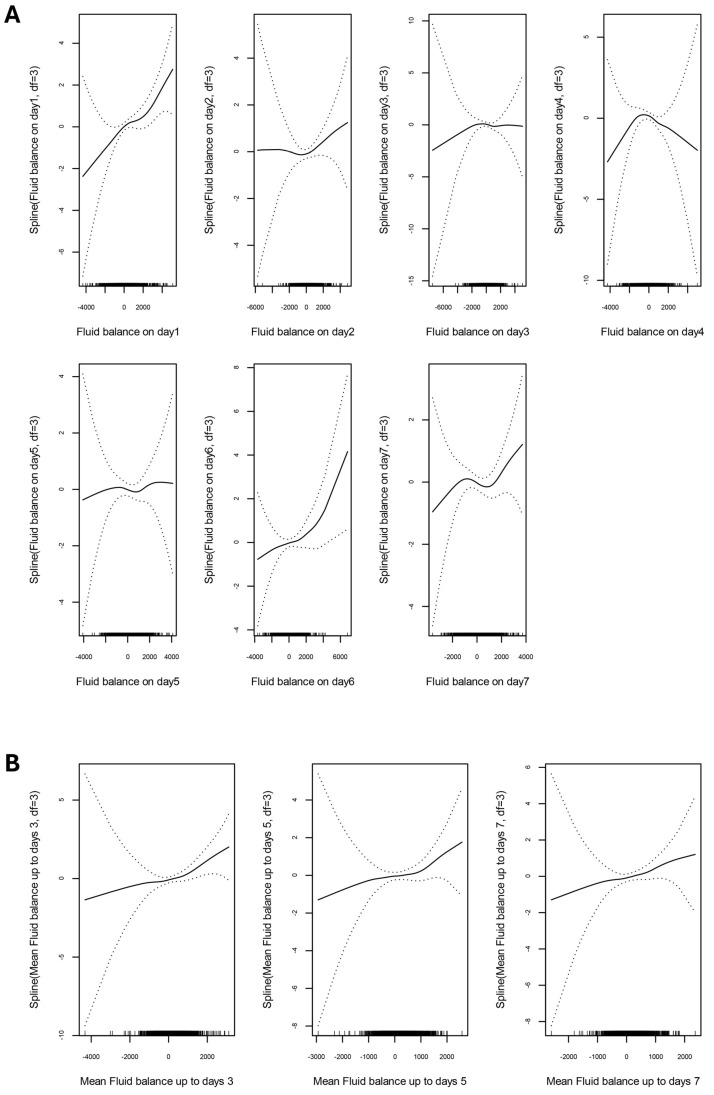
Association between fluid balance and 28-day mortality by using generalized additive model (GAM). (**A**) Daily fluid balance on 28-day mortality. (**B**) Mean fluid balance over extended periods (up to days 3, 5, and 7) on 28-day mortality; GAMs have three degrees of freedom (df). Dashed lines indicate 95% confidence intervals. Vertical bars on the horizontal scale indicate individual observations.

**Figure 3:**
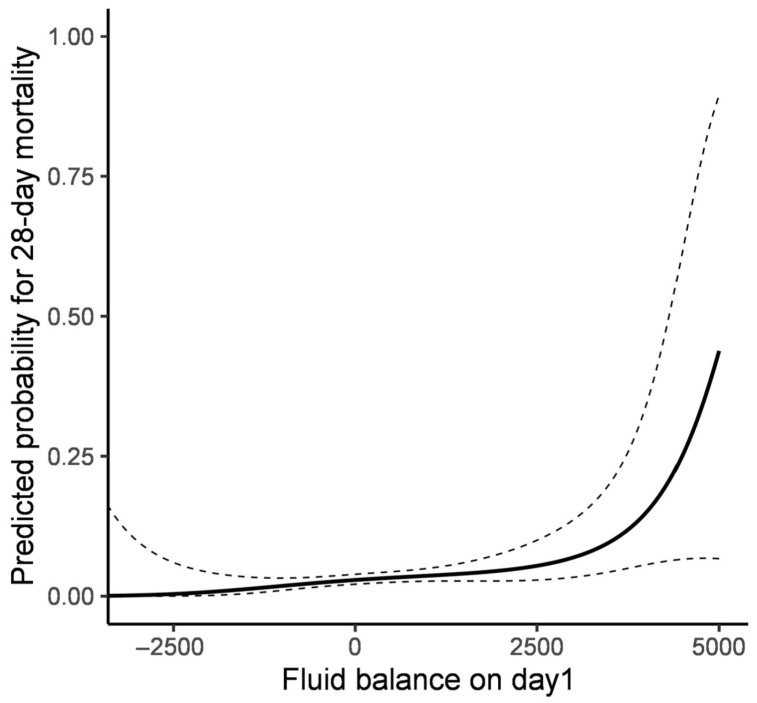
Predicted probability of 28-day mortality according to fluid balance on day1.

**Figure 4:**
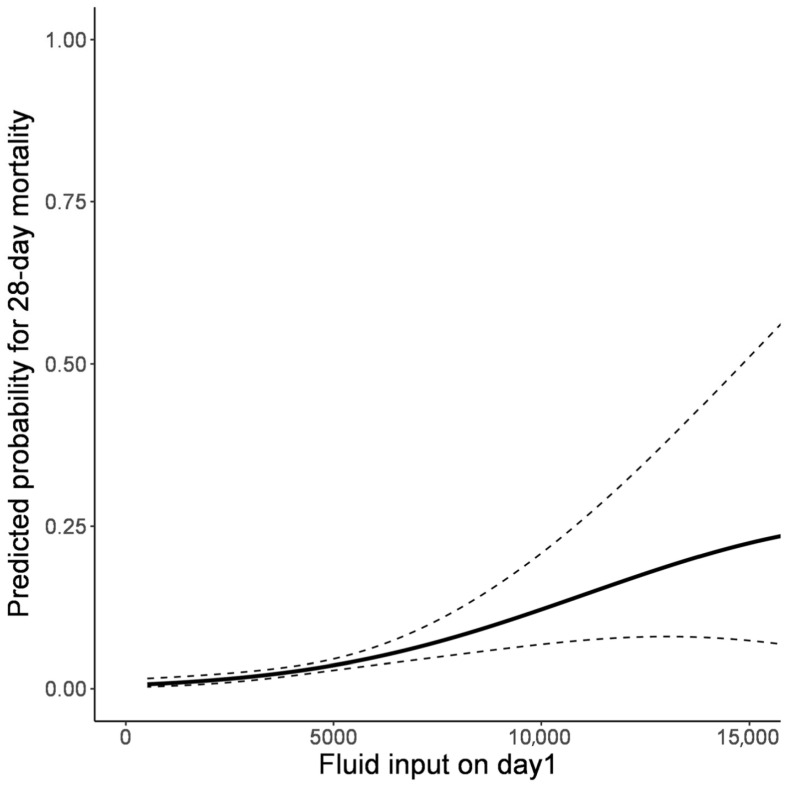
Association between fluid input on day 1 and predictive probability for 28-day mortality.

The GAM analysis demonstrated that among the daily fluid balances, only day 1 and day 6 showed statistically significant associations with 28-day mortality (Figure 2). Further analysis of mean fluid balance over extended periods (up to days 3, 5, and 7) suggested a trend towards increased 28-day mortality with higher fluid balance, although these associations did not reach statistical significance. These findings indicate that early fluid balance, particularly on day 1, may be more crucial in predicting patient outcomes than cumulative fluid balance over time. From the association plot between fluid balance on day 1 and the predicted probability of 28-day mortality, the predicted probability of mortality showed a relatively stable pattern in the negative fluid balance range but demonstrated an increasing trend with more positive fluid balance (Figure 3). Clinical outcomes stratified by fluid balance on day 1 are shown in Table 3. After adjusting for potential confounders including age, underlying comorbidities (malignancy, cerebrovascular disease), and severe neurological impairment (GCS ≤ 12), positive fluid balance on day 1 remained independently associated with increased 28-day mortality (adjusted OR 1.738, 95% CI: 1.013–2.981, *p* = 0.045) (Table 4). As expected, patients with severe neurological impairment (GCS ≤ 12) demonstrated significantly higher mortality risk in the adjusted model (adjusted OR 12.048, 95% CI: 5.682–25.641, *p* < 0.001), confirming that baseline neurological severity is a strong predictor of outcome. The relationship between fluid input and 28-day mortality was further examined using cubic spline in GAM (Figure 4), revealing a progressively increasing probability of 28-day mortality with higher fluid input volumes on day 1. These findings demonstrate a clear association between both increased early fluid balance and cumulative fluid input with higher mortality risk, underscoring the importance of careful fluid management in critically ill neurological patients.

To examine the dose–response relationship and provide clinically applicable thresholds, we performed sensitivity analysis using the following intuitive fluid balance categories: Low (≤−500 mL), Mid (−499 to + 499 mL), and High (≥+500 mL). This analysis demonstrated a clear dose–response pattern, with the High fluid balance group showing significantly increased 28-day mortality risk (adjusted OR 2.896, 95% CI: 1.108–7.568, *p* = 0.026) compared to the Low group, while the Mid group showed a non-significant trend toward increased risk (adjusted OR 2.070, 95% CI: 0.785–5.457, *p* = 0.185). The overall fluid balance effect remained statistically significant (*p* = 0.025) after Bonferroni correction for multiple testing. Subgroup analysis of craniotomy patients revealed an even stronger association, with the High fluid balance group demonstrating substantially elevated mortality risk (adjusted OR 10.559, 95% CI: 1.000–111.543, *p* = 0.050), suggesting that positive fluid balance may be particularly detrimental in surgical patients.

Further analysis examining the association between daily fluid balance, mean fluid balance, and 28-day mortality demonstrated a general trend where increasing positive fluid balance was associated with higher mortality, although statistical significance was only achieved for fluid balance on day 1 (Supplementary Figure S1). This observation reinforces the particular importance of early fluid management in determining patient outcomes, as subsequent daily fluid balances, while showing similar directional trends, did not reach statistical significance in their association with mortality.

## 4. Discussion

This study meticulously analyzed the association between fluid balance and clinical outcomes in a large cohort of neurocritically ill patients. Our key finding was that positive fluid balance and higher fluid input during the early period of ICU admission were significantly associated with increased 28-day mortality. The GAM analysis revealed that fluid management, particularly during the first 24 h of ICU admission, showed the strongest association with mortality outcomes. Notably, non-survivors demonstrated consistently higher daily fluid input throughout the observation period, with the most significant differences observed in the early phase of critical care. Furthermore, multivariate analysis confirmed that fluid balance on day 1 remained independently associated with 28-day mortality after adjusting for potential confounders. These findings underscore the critical importance of careful fluid management in the early phase of neurocritical care, suggesting that early fluid management strategies may significantly impact patient outcomes.

Our findings are largely consistent with previous studies investigating fluid management in neurocritical care [13,14], while also providing novel insights into the timing of fluid administration. Several studies have demonstrated that fluid overload in neurocritically ill patients is associated with poor outcomes [7,15], but most focused on cumulative fluid balance over extended periods. The CENTER-TBI study, one of the largest investigations in traumatic brain injury patients [7], emphasized the importance of avoiding fluid overload, but did not specifically address the critical nature of early fluid management. Our study extends these findings by highlighting the particular significance of fluid balance within the first 24 h of ICU admission. This emphasis on early fluid management represents a crucial advancement in our understanding, as it suggests that initial fluid management decisions may have more profound implications for patient outcomes than previously recognized [16]. Furthermore, while previous studies primarily focused on specific neurological conditions [17,18], our study encompasses a broader spectrum of neurocritical illnesses, providing a more comprehensive perspective on fluid management in neurocritical care.

The physiological mechanisms underlying the association between positive fluid balance and increased mortality in neurocritically ill patients warrant careful consideration [19]. Excessive fluid administration can compromise the already vulnerable blood–brain barrier, potentially exacerbating cerebral edema and elevating intracranial pressure [20]. This is particularly concerning in neurocritical patients, as their normal autoregulatory mechanisms may be impaired, making them more susceptible to fluid-related complications [21]. Furthermore, positive fluid balance can lead to systemic complications such as pulmonary edema, which may further compromise cerebral oxygenation through respiratory dysfunction [22]. The deleterious effects of fluid overload are compounded by the fact that neurocritically ill patients often have altered sodium and osmolar homeostasis, making them particularly sensitive to changes in fluid status [23]. These physiological considerations highlight the importance of maintaining careful fluid balance, especially during the critical early period when the brain is most vulnerable to secondary injury [24].

Based on our findings, careful attention to fluid balance should begin immediately upon ICU admission for neurocritically ill patients, with particular vigilance during the first 24 h [25]. This requires implementation of precise fluid monitoring protocols and regular assessment of fluid status through both clinical and laboratory parameters [26]. The use of advanced hemodynamic monitoring tools may be beneficial in guiding fluid therapy, especially in complex cases with multiple competing physiological demands [27]. Additionally, fluid management strategies should be tailored according to the specific neurological condition, timing of injury, and individual patient characteristics [25]. Regular reassessment of fluid status and timely adjustment of fluid management strategies are essential, as neurocritically ill patients’ needs may change significantly throughout their ICU stay [28].

A critical consideration in interpreting our findings is the potential for confounding by disease severity, as sicker patients may both require more aggressive fluid resuscitation and have higher inherent mortality risk. To address this concern, we adjusted our analysis for multiple severity indicators, including severe neurological impairment, age, and underlying comorbidities. The persistence of the association between positive fluid balance and mortality after these adjustments suggests that the relationship is not simply explained by baseline disease severity or neurological impairment. This indicates that fluid management decisions during the first 24 h of ICU care may have independent prognostic implications beyond what would be expected from patient acuity alone. However, we acknowledge that residual confounding by unmeasured clinical factors cannot be completely excluded in observational studies, and prospective validation with protocolized fluid management strategies is needed to establish causality.

Several limitations of our study should be acknowledged. First, as a single-center retrospective study, our findings may not be fully generalizable to other institutions or patient population. Second, the calculation of fluid balance did not include insensible fluid losses, which could affect the accuracy of our fluid balance measurements. Third, our retrospective analysis could not distinguish between different fluid types and their therapeutic intentions (e.g., osmotic agents for brain edema reduction versus resuscitation fluids for volume expansion), which limits the clinical applicability of our findings regarding fluid management strategies. Fourth, our inability to distinguish between perioperative and post-ICU fluid management represents a significant limitation, as intraoperative fluid administration during anesthesia may confound Day 1 fluid balance calculations. Future prospective studies should incorporate detailed perioperative data collection to separate the effects of surgical versus ICU fluid management on patient outcomes. Fifth, despite our efforts to adjust for confounding factors, the inherent heterogeneity of neurocritically ill patients and the complexity of their care may have introduced unmeasured confounders that could influence our results. Additionally, we were unable to account for the specific timing and rationale behind individual fluid management decisions, which might have provided valuable context for our findings. The borderline statistical significance of our primary finding represents a statistical fragility that limits definitive conclusions, and our results should be interpreted as preliminary evidence requiring validation in larger prospective studies. Future prospective, multicenter studies with standardized protocols for fluid management and more detailed documentation of clinical decision-making processes are needed to validate our findings and establish optimal fluid management strategies for neurocritically ill patients.

## 5. Conclusions

In conclusion, our comprehensive analysis of fluid balance patterns in neurocritically ill patients reveals that early positive fluid balance and higher cumulative fluid input are associated with increased mortality. This relationship is particularly pronounced during the first 24 h of ICU admission, highlighting a critical window for intervention. These findings suggest that careful attention to fluid balance, particularly in the early phase of critical care, should be an essential component of neurocritical care protocols. The development and implementation of evidence-based fluid management strategies, tailored to specific neurological conditions and individual patient characteristics, may significantly improve outcomes in this vulnerable patient population.

## Figures and Tables

**Table 1:** Baseline characteristics of study population.

	Negative Fluid Balance (*n* = 932)	Positive Fluid Balance (*n* = 1254)	*p*-Value
Patient demographics			
Age (year)	56.0 (44.0–66.0)	58.0 (46.0–69.0)	0.003
Sex, male	456 (48.9)	615 (49.0)	0.992
Comorbidities			
Malignancy	602 (64.6)	713 (56.9)	<0.001
Cerebrovascular disease	360 (38.6)	584 (46.6)	<0.001
Hypertension	154 (16.5)	218 (17.4)	0.637
Dyslipidemia	114 (12.2)	154 (12.3)	0.999
Diabetes mellitus	91 (9.8)	121 (9.6)	0.987
Chronic kidney disease	25 (2.7)	46 (3.7)	0.244
Cardiovascular disease	19 (2.0)	24 (1.9)	0.959
Chronic liver disease	7 (0.8)	31 (2.5)	0.004
Behavioral risk factors			
Current alcohol consumption	208 (22.3)	281 (22.4)	0.999
Current smoking	101 (10.8)	149 (11.9)	0.489
Cause of ICU admission			0.001
Brain tumor	491 (52.7)	541 (43.1)	
Intracerebral hemorrhage	101 (10.8)	184 (14.7)	
Subarachnoid hemorrhage	94 (10.1)	141 (11.2)	
Traumatic brain injury	70 (7.5)	121 (9.6)	
Cerebral infarction	32 (3.4)	54 (4.3)	
Elective vascular surgery	49 (5.3)	98 (7.8)	
Central nervous system infection	23 (2.5)	27 (2.2)	
Epilepsy	11 (1.2)	10 (0.8)	
Others	61 (6.5)	78 (6.2)	
APACHE II score on ICU admission	10.0 (7.0–14.0)	11.0 (7.0–16.0)	<0.001
GCS score on ICU admission	14.0 (12.0–15.0)	14.0 (11.0–15.0)	0.108
ICU management			
Mechanical ventilation	426 (45.7)	618 (49.3)	0.107
The duration of mechanical ventilation, day	3.0 (1.0–10.0)	7.0 (2.0–12.0)	<0.001
Continuous renal replacement therapy	12 (1.3)	30 (2.4)	0.088
The duration of using continuous renal replacement therapy, day	5.5 (3.0–9.0)	4.0 (2.0–6.0)	0.207
ICP monitoring	318 (34.1)	404 (32.2)	0.374
The duration of using ICP monitoring, day	8.0 (5.0–11.0)	8.0 (5.0–12.0)	0.697

Data are presented as numbers (%) or medians and interquartile ranges. ICU, intensive care unit; APACHE II, Acute Physiology and Chronic Health Evaluation II; GCS, Glasgow Coma Scale; ICP, intracranial pressure.

**Table 2:** Fluid balance and input according to 28-day mortality.

	Survivor (*n* = 2119)	Non-Survivor (*n* = 67)	*p*-Value
Fluid balance on day 1	156.0 (−432.5–763.5)	480.0 (−104.0–865.5)	0.042
Fluid balance on day 2	110.0 (−456.0–699.5)	222.0 (−379.0–942.0)	0.999
Fluid balance on day 3	120.0 (−415.0–656.5)	57.0 (−387.5–409.0)	0.999
Fluid balance on day 4	150.0 (−380.5–679.6)	48.0 (−450.6–447.5)	0.999
Fluid balance on day 5	155.3 (−359.5–710.0)	108.0 (−446.5–607.0)	0.999
Fluid balance on day 6	174.0 (−384.0–713.0)	323.0 (−265.5–788.0)	0.999
Fluid balance on day 7	110.0 (−440.0–648.0)	−24.0 (−486.9–626.0)	0.999
Mean fluid balance up to day 3	118.2 (−249.8–499.1)	220.0 (−48.9–620.3)	0.219
Mean fluid balance up to day 5	138.4 (−166.3–437.9)	205.8 (−82.7–451.1)	0.996
Mean fluid balance up to day 7	128.9 (−126.6–405.4)	238.6 (−56.9–426.8)	0.438
Fluid input on day 1	3978.0 (3152.5–4896.0)	4444.0 (3668.5–6491.8)	0.007
Fluid input on day 2	3900.0 (3021.0–4984.0)	4407.0 (3559.0–5304.0)	0.112
Fluid input on day 3	3689.0 (2816.5–4767.0)	4476.0 (3390.5–5686.5)	0.007
Fluid input on day 4	3470.0 (2603.5–4555.5)	4404.0 (3030.0–5690.4)	<0.001
Fluid input on day 5	3280.0 (2510.0–4313.9)	4105.0 (3018.0–5279.0)	0.007
Fluid input on day 6	3145.0 (2350.0–4205.0)	3986.0 (2561.0–5038.5)	0.007
Fluid input on day 7	2933.0 (2029.0–3959.0)	3410.0 (2289.5–4394.5)	0.147
Mean fluid input up to day 3	3905.3 (3141.3–4817.8)	4689.0 (3671.7–5357.4)	<0.001
Mean fluid input up to day 5	3705.2 (3006.4–4605.3)	4644.6 (3498.5–5343.8)	<0.001
Mean fluid input up to day 7	3513.2 (2833.6–4417.7)	4348.6 (3308.4–5210.1)	<0.001

Data were represented as mL. *p*-values were corrected using Bonferroni’ method due to multiple testing.

**Table 3:** Clinical outcomes according to fluid balance on day 1.

	Negative Fluid Balance (*n* = 932)	Positive Fluid Balance (*n* = 1254)	*p*-Value
Ventilator-free days	25.0 (17.0–27.0)	21.0 (9.0–26.0)	<0.001
ICU length of stay	3.9 (2.1–7.6)	4.0 (2.0–9.0)	0.793
Hospital length of stay	29.9 (16.8–47.5)	26.8 (14.5–46.0)	0.007
ICU mortality	24 (2.6)	56 (4.5)	0.027
28-day mortality	20 (2.1)	47 (3.7)	0.043
In-hospital mortality	41 (4.4)	99 (7.9)	0.001

ICU, intensive care unit.

**Table 4:** Independent predictors of 28-day mortality: results from multivariable logistic regression.

Variables	Adjusted OR (95% CI)	*p*-Value
Fluid balance on day1		
Positive value group	1.738 (1.013–2.981)	0.045
Zero or Negative value group	Ref	
Age	1.002 (0.986–1.0118)	0.802
Malignancy	0.732 (0.419–1.278)	0.272
Cerebrovascular disease	0.616 (0.349–1.087)	0.095
GCS ≤ 12	12.048 (5.682–25.641)	<0.001

OR, odd ratio; CI confidence interval; Ref, reference category.

## Data Availability

Regarding data availability, our data are available on the Harvard Dataverse Network (http://doi.org/10.7910/DVN/2XTZ6Z, accessed on 10 February 2025).

## Supplementary Materials

The following supporting information can be downloaded at: https://www.mdpi.com/article/10.3390/jcm14155518/s1, Figure S1: Association of daily fluid balance and mean fluid balance with 28-day mortality: Forest plot analysis. OR odd ratio, CI confidence interval.

## Abbreviations

ICU	intensive care unit
ICP	intracranial pressure
GAM	generalized additive model
OR	odds ratio
CI	confidence interval
APACHE	Acute Physiology and Chronic Health Evaluation
GCS	Glasgow Coma Scale
