# Supplementary material for: Early Positive Fluid Balance Associates with Increased Mortality in Neurological Critically Ill Patients: A 10-Year Cohort Study
Source: J Clin Med. 2025 Aug 5;14(15):5518. doi: 10.3390/jcm14155518 (PMC12347425; doi:10.3390/jcm14155518)

Supplementary figure 1. Association of daily fluid balance and mean fluid balance with 28-day mortality: Forest plot analysis. *OR* odd ratio, *CI* confidence interval

Variable	Freq/Patients	Prevalence	OR (95% CI), P value
Net Balance			
day1			
more than 0	47 / 1252	3.754	1.78 (1.05 to 3.03) 0.0326
equal or less than 0	20 / 934	2.141	
day2			
more than 0	41 / 1216	3.372	1.27 (0.77 to 2.09) 0.353
equal or less than 0	26 / 970	2.68	
day3			
more than 0	31 / 1229	2.522	0.90 (0.55 to 1.47) 0.677
equal or less than 0	36 / 957	3.762	
day4			
more than 0	31 / 1263	2.454	0.84 (0.52 to 1.38) 0.496
equal or less than 0	36 / 923	3.9	
day5			
more than 0	39 / 1279	3.049	0.99 (0.60 to 1.62) 0.96
equal or less than 0	28 / 907	3.087	
day6			
more than 0	46 / 1281	3.591	1.57 (0.93 to 2.65) 0.0921
equal or less than 0	21 / 905	2.32	
day7			
more than 0	33 / 1220	2.705	0.76 (0.47 to 1.24) 0.274
equal or less than 0	34 / 966	3.52	
Average Net Balance			
day3			
more than 0	45 / 1280	3.516	1.46 (0.87 to 2.46) 0.148
equal or less than 0	22 / 906	2.428	
day5			
more than 0	40 / 1346	2.972	0.92 (0.56 to 1.51) 0.749
equal or less than 0	27 / 840	3.214	
day7			
more than 0	47 / 1373	3.423	1.40 (0.83 to 2.39) 0.209
equal or less than 0	20 / 813	2.46	

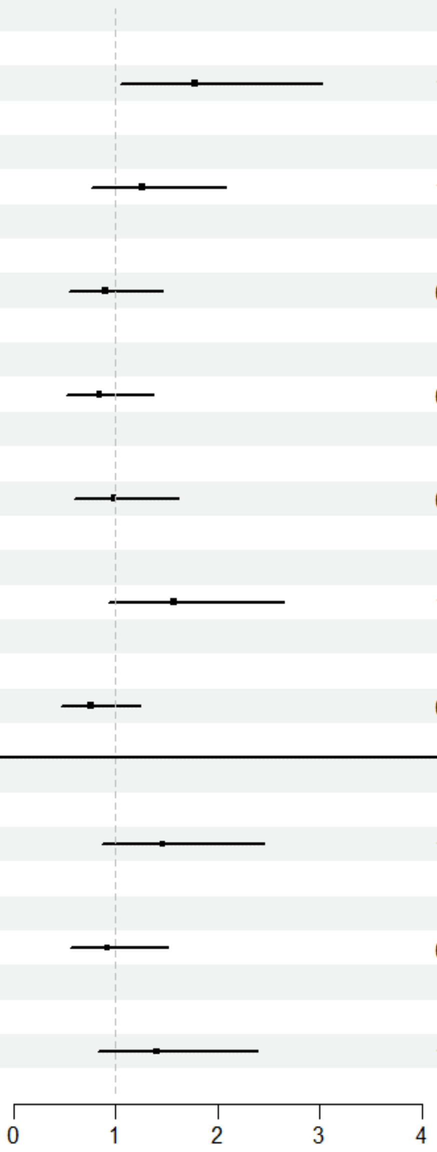

Supplement: Supplementary file 1 [file jcm-14-05518-s001.zip › Supplementary figure 1.pdf]
